# Validity and Reliability of Smartphone App for Evaluating Postural Adjustments during Step Initiation

**DOI:** 10.3390/s22082935

**Published:** 2022-04-12

**Authors:** Anderson Antunes da Costa Moraes, Manuela Brito Duarte, Eduardo Veloso Ferreira, Gizele Cristina da Silva Almeida, Enzo Gabriel da Rocha Santos, Gustavo Henrique Lima Pinto, Paulo Rui de Oliveira, César Ferreira Amorim, André dos Santos Cabral, Anselmo de Athayde Costa e Silva, Givago Silva Souza, Bianca Callegari

**Affiliations:** 1 Human Motricity Studies Laboratory, Av. Generalíssimo Deodoro 01, Belém 66073-000, PA, Brazil; antunesanderson@hotmail.com (A.A.d.C.M.); manubritd@gmail.com (M.B.D.); eduadocvf@gmail.com (E.V.F.); g.c.fisioterapia@gmail.com (G.C.d.S.A.); 2 Institute of Exact and Natural Sciences, Federal University of Pará, R. Augusto Corrêa, 01, Belém 66093-020, PA, Brazil; enzogabrielrocha29@gmail.com (E.G.d.R.S.); gpinto@ufpa.br (G.H.L.P.); 3 Doctoral and Master’s Program in Physical Therapy, UNICID, 448/475 Cesário Galeno St., São Paulo 03071-000, SP, Brazil; paulo.oliveira@unicid.edu.br (P.R.d.O.); cesar@emgsystem.com (C.F.A.); 4 Département des Sciences de la Santé, Programme de Physiothérapie de L’université McGill Offert en Extension à l’UQAC, Saguenay, QC G7H 2B1,Canada; 5 Physical Therapy and Neuroscience Departments, Wertheims’ Colleges of Nursing and Health Sciences and Medicine, Florida International University (FIU), Miami, FL 33199, USA; 6 Center for Biological and Health Sciences, Pará State University, Tv. Perebebuí, 2623—Marco, Belém 66087-662, PA, Brazil; andre.cabral@uepa.br; 7 Postgraduate Program in Movement Science, Federal University of Pará, Av. Generalíssimo Deodoro 01, Belém 66073-000, PA, Brazil; anselmocs@ufpa.br; 8 Institute of Biological Sciences, Federal University of Pará, R. Augusto Corrêa 01, Belém 66075-110, PA, Brazil; givagosouza@ufpa.br; 9 Tropical Medicine Nucleus, Federal University of Pará, Avenida Generalíssimo Deodoro 92, Belém 66055-240, PA, Brazil

**Keywords:** anticipatory postural adjustments, smartphone, gait initiation

## Abstract

The evaluation of anticipatory postural adjustments (APAs) requires high-cost and complex handling systems, only available at research laboratories. New alternative methods are being developed in this field, on the other hand, to solve this issue and allow applicability in clinic, sport and hospital environments. The objective of this study was to validate an app for mobile devices to measure the APAs during gait initiation by comparing the signals obtained from cell phones using the *Momentum* app with measurements made by a kinematic system. The center-of-mass accelerations of a total of 20 healthy subjects were measured by the above app, which read the inertial sensors of the smartphones, and by kinematics, with a reflective marker positioned on their lumbar spine. The subjects took a step forward after hearing a command from an experimenter. The variables of the anticipatory phase, prior to the heel-off and the step phase, were measured. In the anticipatory phase, the linear correlation of all variables measured by the two measurement techniques was significant and indicated a high correlation between the devices (APA_onset_: r = 0.95, *p* < 0.0001; APA_amp_: r = 0.71, *p* = 0.003, and PEAK_time_: r = 0.95, *p* < 0.0001). The linear correlation between the two measurement techniques for the step phase variables measured by ques was also significant (STEP_interval_: r = 0.56, *p* = 0.008; STEP_peak1_: r = 0.79, *p* < 0.0001; and STEP_peak2_: r = 0.64, *p* < 0.0001). The Bland–Altman graphs indicated agreement between instruments with similar behavior as well as subjects within confidence limits and low dispersion. Thus, using the *Momentum* cell phone application is valid for the assessment of APAs during gait initiation compared to the gold standard instrument (kinematics), proving to be a useful, less complex, and less costly alternative for the assessment of healthy individuals.

## 1. Introduction

The central nervous system uses postural adjustment strategies to neutralize balance disturbances by anticipatory postural adjustments (APAs) and compensatory postural adjustments. These aim to maintain the posture in case of a disturbance or an imbalance, helping to control balance and perform everyday life activities, such as [1,2].

APAs are characterized by previous center of pressure (COP) movements in the opposite direction to the disturbance and by the pre-activation of the musculature linked to the synergy and kinematic chain associated with the disturbance. To allow the determination of APAs, a sufficiently high degree of destabilization is necessary to cause considerable displacement of the center of mass (COM) [3]. APAs arise as effective mechanical mechanisms in maintaining the vertical posture in humans. In this regard, many studies have already been dedicated to the identification and quantification of APAs. However, more recent data indicate that changes in the levels of the activation of the postural muscles caused by APAs in anticipation of a self-initiated action are generally seen starting approximately 100 ms before the onset of the action [4,5].

On gait initiation, it is known that a correct sequence of movements is required for step preparation and execution [6,7]. Therefore, APAs precede the beginning of voluntary gait movements to reduce the effect of external body disturbances. In this case, APAs act to accelerate the COM forward and laterally toward the support foot, displacing the COP posteriorly and toward the swing leg [6].

With a lateral COP shift, the feet are actively raised, and the COP moves toward the heels. This strategy moves the COM in front of the COP, causing the body to fall forward, supported only by the supporting foot. Thus, the first phase of the COP shift toward the support foot is known as the “unbalance phase” and the second as the “discharge phase” [8,9]. In this regard, Ellmers et al. (2020) explained that there are two distinct phases of the postural adjustment before the start of a step. In the first phase, the APAs involve the posterior and lateral displacements of the COP toward the swing foot/step, and in the second, there is a weight change, with a lateral change in the COP toward the support foot. Following this weight change, the COM is repositioned above the support foot/support, and the balance/step foot is unloaded and free to start walking [8].

Currently, evaluation of APAs requires systems of high cost and complex handling, such as surface electromyography, a force platform for measuring the COP, and kinematic analysis using cameras. This restricts the measurements and the assessments to research laboratory environments, limiting their applicability in clinics and hospitals [10].

However, in recent years, new methods have been proposed owing to their low cost and high practicality for assessing APAs at gait initiation [11,12]. Currently, there is an increase in the development and use of modern applications (apps) in cell phones, known as smartphones, which incorporate hardware components that allow the assessment of body balance. These devices are available to a large part of the population, with many of them having built-in inertial sensors, for which some studies have analyzed the signals registered by cell phone sensors in static balance [13,14] to measure spatiotemporal gait parameters [15,16].

Although the development of apps to assess balance, gait, and clinical applications in patients is growing, based on the literature, studies that evaluate APAs at gait initiation using smartphone apps are lacking, reinforcing the need for the development of research in this area.

In this regard, this research seeks to propose and validate an app for mobile devices to measure the APAs during gait initiation. Specifically, we are proposing and validating a new mobile app to evaluate step initiation based on the mobile’s accelerometer signal. Additionally, its objective is to validate the cell phone signals generated by the measurements obtained using a kinematic system. Our hypothesis is that the data recorded with smartphones using this app can achieve reliable and gold-standard evaluations of the postural adjustments at gait initiation.

## 2. Materials and Methods

### 2.1. Subjects

Twenty healthy individuals of both sexes aged between 18 and 40 years old and with body mass index values within the limits of 18.5–24.9 agreed to participate in the research and signed their informed consent. All procedures used in this research were approved by the Ethics Committee of the Federal University of Pará, Health Sciences Institute under reference number 3.773.655 CAEE: 25667919.2.0000.0018. The participants were recruited for convenience and the screening and research evaluations were performed at the Human Movement Laboratory (Laboratório do Movimento Humano—LEMOH) of the Federal University of Pará, located at Avenida Generalíssimo Deodoro, n 01 CEP 66055-240, Belém-PA.

The sample size was calculated using 80% statistical power and 95% confidence interval. The mean and standard deviation for the APA_onset_ (s) was estimated in a pilot study performed in the first 6 subjects in two sessions. The mean difference between sessions was achieved as 0.084978 ± 0.09863 s. A required sample size of 13 individuals was calculated.

No participant referred neurological or vestibular complaints, and these potential disorders were screened using index-index and Romberg tests [17]; altered gait and orthopedic trauma were excluded from the research, and individuals with corrected vision wore glasses during the experiment.

### 2.2. Instruments

To measure and record the COM accelerations in the three axes during gait initiation, two instruments were used: a three-dimensional system with three cameras (SIMI Reality Motion Systems 3D, Unterschleißheim, Germany) and a mobile device (Android A10s, Samsung, Seoul, Korea) with accelerometer sensors and 2-GHz and 1.5-GHz speed Octa Core processors. Filming was conducted at a sampling frequency of 120 Hz. An app called “*Momentum*”, installed on the mobile device with an Android platform in the Java language, was used for registration to assess the accelerations of body oscillations. The basic concept of the developed code was the use of the Android SDK library, which contains functions that allow access to sensors available on a mobile device. The app stored the data measured by the accelerometer of the mobile device at a sampling rate of 50 Hz. The output of the app is designed to report oscillations in three axes: vertical, anteroposterior, and mediolateral.

Finally, to mark the contact of the foot with the ground, foot switch (FS) sensors were used, with an acquisition rate of 2000 Hz. Each sensor consisted of two round plates of 2 cm in diameter (EMG System do Brasil, Ltd.a., São José, SP, Brazil) composed of a conductive and moldable material separated by a soft nonconductive material.

### 2.3. Experimental Protocol

**Figure 1:**
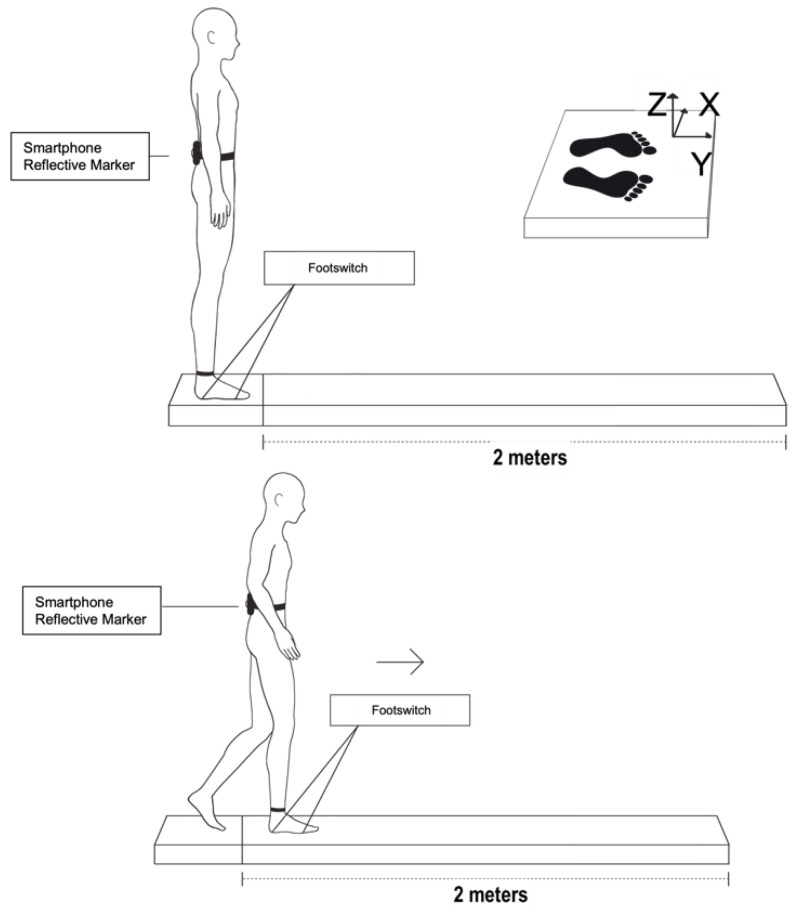
Subject standing on platform with cell phone and reflective marker at L5 and FSs at base of calcaneus and head of second metatarsal. Subject starting gait with right leg by 2-m walkway.

The subjects remained standing, erect, and barefoot in a comfortable position, with their arms at their sides. The FSs were fixed on the right forefoot and hindfoot of each individual to mark the moment when the heel leaves the ground and the complete demarcation of the step support phase. The mobile device was held in the region of the fifth vertebra of the lumbar spine (L5) by a neoprene strap, and a reflective marker was placed on the cell phone to acquire the data from the kinematics system (Figure 1). We chose to use only one marker to measure the acceleration of a single point, equivalent to the location of the smartphone.

**Figure 2:**
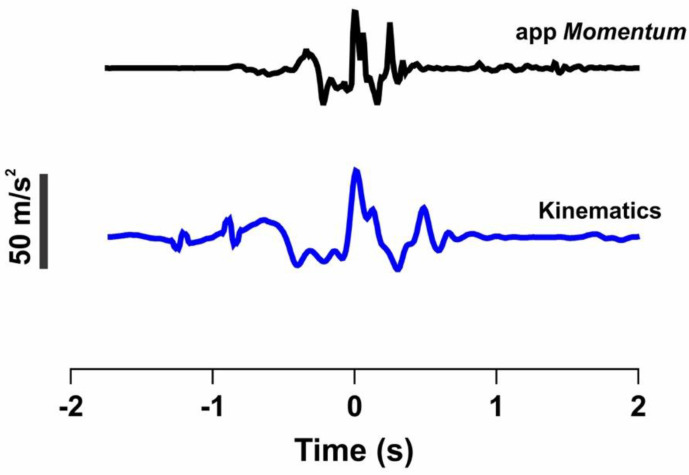
Vertical acceleration signals registered by *Momentum* app (black line) and by kinematics (blue line) during vertical jump. Dotted line represents peak acceleration on this axis, corresponding to impact with ground, which is used for synchronization. Acc: Acceleration.

Before starting to walk, the subjects were asked to jump on the spot, through the signal peak, for synchronization among all research collection instruments, as shown in Figure 2.

After this step, the subjects remained standing at the beginning of the 2-meter walkway for the initiation of the experiment. The foot position for each trial was controlled by asking the subjects to place their feet on the marks drawn on the walkway. The mediolateral distance between the heels was 6 cm. At the beginning of each trial, the subjects focused on a mark at eye level on the wall, at a distance of 3 m. The subjects were asked to step forward on hearing a command from an experimenter. The command was given at random, without prior warning. Before recording the data, the subjects were asked to perform a cycle to confirm that they had understood the instructions correctly. Each subject performed ten trials, always starting with the right lower limb, where the FS was fixed.

To analyze the reliability of the protocol, the entire experiment was performed twice, on alternate days, with a week between them.

### 2.4. Signal Processing

All data obtained using the equipment were synchronized for comparison. Data synchronization and offline analysis were performed using a MATLAB program (MathWorks, Natick, MA, USA) and conducted from the initial jump.

**Figure 3:**
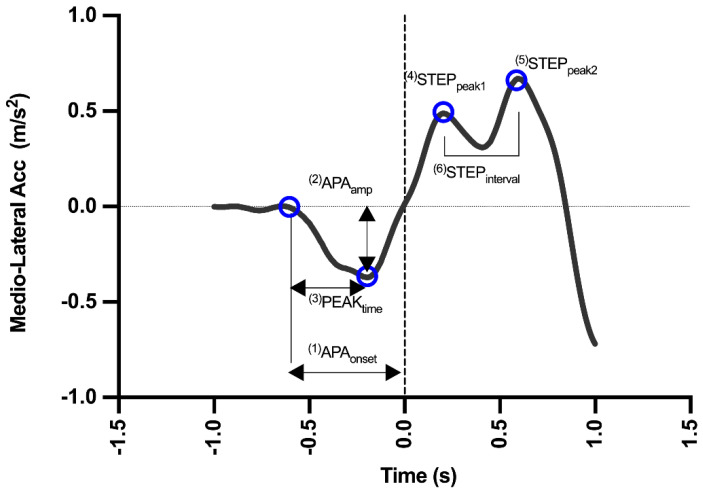
Mediolateral (ML) acceleration curve extracted from the kinematic signal of one subject. The variables included in the method are expressed by numbers and blue circles in the graph (meaning of each variable are explained in method session). The dashed line represents moment when heel leaves ground.

An average of ten trials was used for all calculations. The moment of heel-off was defined by the FS. After defining this moment, the trials were conducted within each series for each subject. Subsequently, the COM accelerations in the mediolateral (ML) direction, extracted from the records of the measuring instruments, were analyzed. ML axis was chosen, as it represented the main axis where APAs occur during step initiation [18]. Raw data coordinates from all signals on the mediolateral axis were generated from a video analysis and the *Momentum* app and subsequently filtered with a 10-Hz second-order low-pass Butterworth filter. Filtering was applied as published by Martinez-Mendez, Sekine and Tamura [18], by performing the power spectrum analysis of the signals, and the same cut-off frequency was employed for all the signals. Figure 3 presents a typical acceleration waveform expected for both devices used in the present method. The following are the calculated and exported step parameters:

(1) APA_onset_: this is the APA latency, the moment when the first mediolateral deviation that exceeds two standard deviations (SDs) above the baseline occurs;
(2) APA_amp_: this is the maximum mediolateral displacement of the COM before the heel-off.
(3) PEAK_time_: this is the time taken to reach the maximum amplitude.
(4) STEP_peak1_: this is the first mediolateral deviation after the heel-off;
(5) STEP_peak2_: this is the second mediolateral deviation after the heel-off;
(6) STEP_interval_: this is the interval between two mid-side peaks.

### 2.5. Statistical Analysis

Statistical analysis was conducted using GraphPad PRISM 9 software. The Shapiro–Wilk test confirmed the normality of the data distribution, and data description was presented using boxplot graphs for each parameter. A boxplot displays the minimum value, lower quartile (25th percentile), median, upper quartile (75th percentile), and maximum value. The median was represented inside the boxplot. For the validation of the *Momentum* app, the measured variables from the different devices were correlated by Pearson correlation tests (r) when the data were parametric (APA_amp_ and PEAK_time_) or Spearman (APA_onset_, STEP_peak1_, STEP_peak2_, and STEP_interval_). From the correlation tests, the point-to-point agreement between the systems was estimated, and the *r* values and the confidence intervals were determined. Pearson’s correlation coefficients (r) were interpreted with magnitude thresholds of 0–0.1: trivial, 0.1–0.3: small, 0.3–0.5: moderate, 0.5–0.7: large, 0.7–0.9: very large, and 0.9–1.0: almost perfect [19]. Subsequently, the Bland–Altman graphs with a 95% limit of agreement (mean ± 2 SD) were plotted to compare the equipment values. Paired Student’s t-tests were conducted for the variables with a normal distribution (APA_amp_ and PEAK_time_) and Wilcoxon tests for nonparametric variables (APA_onset_, STEP_peak1_, STEP_peak2_, and STEP_interval_). The level of significance was defined as *p* < 0.05. The reliability between two test sessions was calculated from the mean values of the trials recorded during each session. Subsequently, for reliability, each intraclass correlation coefficient (ICC), with a bidirectional mixed model and the absolute agreement with 95% confidence intervals (CI), was calculated to determine the absolute reliability. The ICCs were interpreted based on the method of Shrout and Fleiss [19], where ICC ≥ 0.75 indicates excellent correlation, ICC = 0.74–0.4 indicates reasonable to high correlation, and ICC ≤ 0.39 indicates poor correlation [20,21]. The level of significance was defined as *p* < 0.05.

## 3. Results

**Figure 4:**
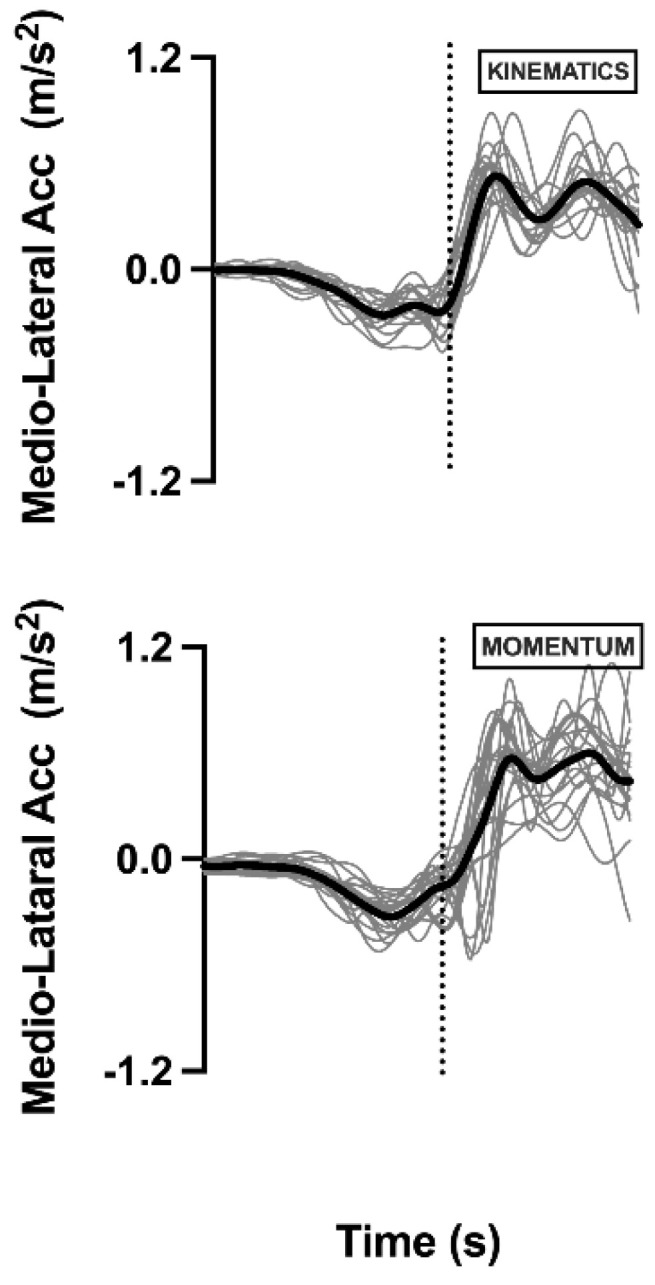
COM accelerations of each subject and resultant from first session. Data from kinematics and *Momentum* app are represented. Thick line represents average resulting from 20 subjects. Dashed line represents heel-off moment. (ML: Mediolateral).

Figure 4 shows the resultant (from the ten attempts) of the medio lateral acceleration of the COM for each measuring device. This is a result figure presenting the similar characteristics between subjects and methods. Based on the images, in both the kinematics system and *Momentum* app, the COM presents the same behavior, indicating that in this study, both techniques characterized the events of the subjects similarly.

**Figure 5:**
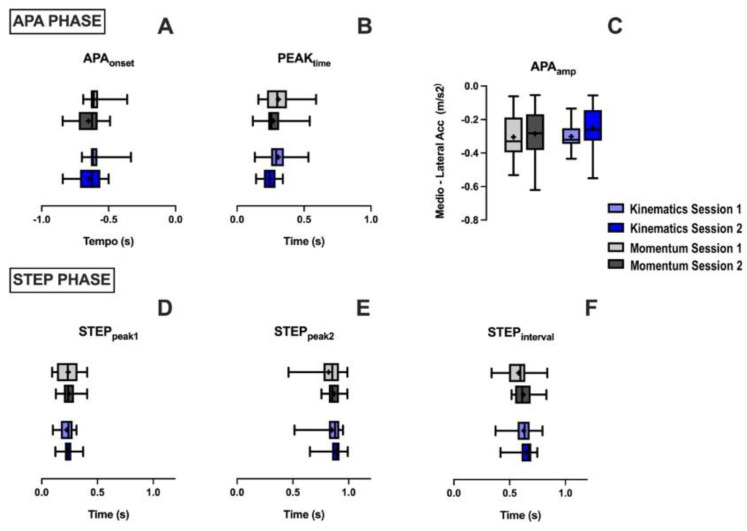
Analysis of mean and SD of subjects in first and second sessions, with both instruments: Kinematics and *Momentum* app. Top part of graph presents the anticipatory variables, and those below are step variables after heel-off. (**A**): APA_onset_; (**B**): PEAK_time_; (**C**): APA_amp_; (**D**): STEP_peak1_; (**E**): STEP_peak2_, and (**F**): STEP_interval_.

The mean of each studied variable is shown in Figure 5. Based on the results, no statistically significant differences are observed in the sessions of both instruments for the anticipatory (before the heel-off) and step variables (after the heel-off).

### 3.1. Validation

**Figure 6:**
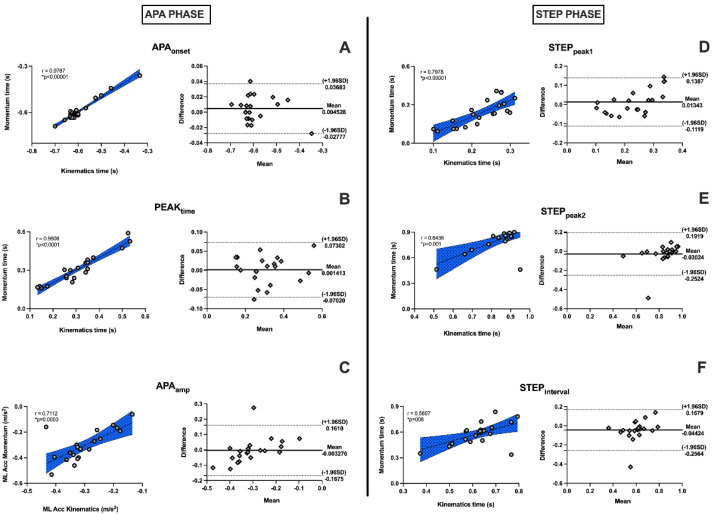
Linear correlation graphs and Bland–Altman correlation graphs showing high correlation between assessment instruments. r ≥ 0.7 represents very high correlation, and asterisk (*) represents values that present statistical significance (*p* ≤ 0.05). Anticipatory variables are shown to left of graph, and step variables, after heel-off, to right. (**A**): APA_onset_; (**B**): PEAK_time_; (**C**): APA_amp_; (**D**): STEP_peak1_; (**E**): STEP_peak2_, and (**F**): STEP_interval_.

The Bland–Altman graphs (Figure 6) showed agreement between the instruments in both the APA and step phase, given that the means of the differences were close to zero, for equipment with similar behaviors and with subjects within confidence limits, presenting little dispersion. This demonstrates that the *Momentum* app as well as the kinematics equipment, which is the gold standard, could assess the postural adjustments at gait initiation. The linear correlation of all variables was significant and presented an r ≥ 0.7, indicating an extremely high correlation between the devices. An exception was ACC_peak_, which presented a r = 0.65, indicating a high correlation (Figure 6).

### 3.2. Reliability

The ICCs between the sessions recorded with kinematics and *Momentum* did not present statistically significant results, and thus, indicate not good reliability, neither for the anticipatory variables nor for the step variables. One exception was the STEP_peak1_ variable, which showed reasonable to high reliability (ICC 0.709) and *p* = 0.04 (Table 1).

## 4. Discussion

The present study aimed to validate an app for mobile devices to measure APAs during gait initiation by comparing the measurements obtained using a cell phone with the signals from a camera system, considered as the gold standard. We hypothesized that the data obtained using smartphones with the app could assess postural adjustments at gait initiation, with a validity relative to the gold standard and with reliable data collection during the sessions. This hypothesis was partially acceptable, because although the results validated the cell phone measurements for both the anticipatory and step variables, the reliability between the sessions was not confirmed. Only the STEP_peak1_ variable had reasonable to high reliability.

Previous studies on human movement have already confirmed the validity of the measurements obtained from smartphone accelerometers. Most studies only included static balance and spatiotemporal gait parameters protocols [22,23,24].

Roeing et al. [22] presented a systematic review of 13 studies that assessed the balance and risk of falling in the elderly and tested the validity and reliability of cell phone measurements with gold standard technologies. All studies in the review were successful in assessing the validity and reliability of smartphone measurements [22]. Fiems et al. [25] analyzed the use of the “Sway Balance” mobile app in the assessment of postural sways in individuals with Parkinson’s disease (PD). They demonstrated the concurrent validity with strong test–retest reliability of the measurements obtained using inertial sensors, proposing the use of mobile apps for this purpose. Polechonski et al. [24] demonstrated that smartphones with a gyroscope have the potential to perform accurate postural balance assessments as an alternative to expensive and specialized equipment. They concluded that reliable measurements can be obtained with a smartphone and a force platform for measuring lateral and anteroposterior oscillations.

A recent study by Arcuria et al. [15] sought to develop an app capable of assessing static and dynamic balance in patients with cerebellar ataxias. This app worked through a device (smartphone) placed at the height of the sternum and measured the body sway using a triaxial accelerometer. Forty patients with cerebellar ataxia and eighty healthy individuals were studied to determine their static and dynamic balance, using the “Berg balance scale”, a “scale for the assessment and rating of ataxia”, and a force platform. The authors concluded that the intra-examiner and test–retest reliability of the measurements, estimated by the ICC, were excellent. This indicated that the app is an easy, reliable, and valid assessment system to quantify trunk sway in a static position and while walking.

Although the possibility of using mobile technology in the assessment of balance and stability has been recognized [24,26], a comparison of the results of the present study with the current literature is unfeasible. This is because until now, few studies assessed APAs in the initiation of gait using accelerometers, and our potential contribution is to perform this assessment with a cell phone (Table 2), which makes this research pioneering.

Only the spatiotemporal parameters of gait have been validated based on the measurements obtained with a smartphone.

Abou et al. [26] conducted a systematic review to summarize the validity and reliability of smartphone apps to assess gait, balance, and falls in PD. They concluded that smartphone apps demonstrated strong validity and reliability for assessing gait and balance and detecting the freezing of gait in individuals with PD. However, the ability of smartphone apps to predict future falls in this population was inconclusive and deserves further exploration. In this regard, Fiems et al. [23] found that the SWAY mobile app alone does not demonstrate higher accuracy in predicting future falls due to PD than the knowledge of the history of falls of an individual.

Su et al. [27] demonstrated excellent validity of a mobile app in gait assessment of individuals with PD, with high correlations observed between the stride time and the stride time variability (r = 0.98–0.99, *p* < 0.001). In this regard, Clavijo-Buendia et al. [28] analyzed the validity and test–retest reliability of the free mobile app RUNZI^®^ in people with mild to moderate PD, and found that the smartphone can perform spatiotemporal analysis of gait and complement conventional assessment methods. Tang et al. [29] reported a strong correlation between a smartphone app and a research-level accelerometer that measures gait and detects gait freeze.

Although some of the previous studies have shown reliability of the measurements recorded by smartphone accelerometers, the present study did not confirm them. The low reliability found in the kinematics records, considered as the gold standard, is probably related to the experimental protocol used in this research, in which spatiotemporal gait variables such as step length and duration, speed, or reaction time to the command were uncontrolled. Accordingly, the individuals displayed different ways of starting gait, resulting in low replicability. However, controlling the spatiotemporal parameters of gait was not an option in the study, given that there are no international standards for the parameterization of protocols to start a step and visualize APA events.

This study presents some limitations, mainly related to the lack of evidence in the use of accelerometers built into smartphones for the assessment of APAs, thus making it difficult to compare our results with the literature. In addition, investigation concerning the test–retest reliability is needed, so we believe it is necessary to parameterize gait during the execution of the experiment with the analysis of the speed, time, and cadence between sessions, to achieve improved analysis, and thus, satisfactory reliability. Furthermore, it is suggested that future research implements assessment protocols with smartphones in unhealthy individuals, with the aim of achieving better applicability during the treatment of patients.

Overall, the present study concluded that the use of the *Momentum* app is valid for the assessment of the APAs during gait initiation compared with the gold standard instrument, kinematics. Thus, as beneficial points, cell phones proved to be a useful, less complex, and less expensive alternative to kinematics for the evaluation of healthy individuals. However, there is a need for more research aimed at analyzing the test–retest reliability in the use of smartphones for the assessment of APAs, because only the STEP_peak1_ variable shows satisfactory reliability among the sessions. Therefore, the outcomes cannot be generalized.

## Figures and Tables

**Table 1:** Intrasession intraclass correlation coefficient (ICC) results of all variables analyzed by kinematics and *Momentum* app. Asterisk (*) represents values that present statistical significance (*p* ≤ 0.05).

Variable	ICC	Lower Limit	Upper Limit	F	df1	Df2	*p*-Value
**APA_onset_**							
Kinematics	0.014	−1.072	0.570	1.016	20	20.196	0.485
*Momentum*	0.185	−0.633	0.635	1.279	20	20.506	0.291
**APA_amp_**							
Kinematics	0.266	−0.633	0.689	1.397	20	20.959	0.226
*Momentum*	0.451	−0.381	0.779	1.795	20	20.347	0.098
**PEAK_time_**							
Kinematics	−0.284	−2.151	0.484	0.742	20	9.31	0.725
*Momentum*	0.002	−1.343	0.597	1.021	20	20.105	0.481
**STEP_peak1_**							
Kinematics	0.672	0.200	0.866	3.031	20	20.829	0.007
*Momentum*	0.709	0.274	0.882	3.347	20	20.237	0.004 *
**STEP_peak2_**							
Kinematics	0.148	−1.001	0.647	1.179	20	20.660	0.355
*Momentum*	0.384	−0.406	0.742	1.674	20	20.829	0.125
**STEP_interval_**							
Kinematics	0.380	−0.508	0.747	1.614	20	20.772	0.142
*Momentum*	0.402	−0.387	0.751	1.711	20	20.953	0.115

**Table 2:** Summary of literature using Accelerometers for APAs assessments during gait initiation.

Study	Device	Variable	Validity Comfirmed
Present	Mobile	APA_onset_; PEAK_time_; APA_amp_; STEP_peak1_; STEP_peak2_, STEP_interval_	yes
Mancini et al., 2016	IMU	APA duration; APA amplitude. Step spatiotemporal parameters	yes
Martinez-Mendez et al., 2011	IMU	APA duration; APA amplitude.	In part

## Data Availability

Please contact the corresponding author.
